# TMIGD2 as a potential therapeutic target in glioma patients

**DOI:** 10.3389/fimmu.2023.1173518

**Published:** 2023-05-16

**Authors:** Chaimae Boulhen, Saadia AIT SSI, Hamza Benthami, Ibtissam Razzouki, Abdelhakim Lakhdar, Mehdi Karkouri, Abdallah Badou

**Affiliations:** ^1^ Immuno-Genetics and Human Pathology Laboratory, Faculty of Medicine and Pharmacy, Hassan II University, Casablanca, Morocco; ^2^ Laboratory of Pathological Anatomy, University Hospital Center (CHU) Ibn Rochd, Hassan II University, Casablanca, Morocco; ^3^ Department of Neurosurgery, Faculty of Medicine and Pharmacy, University of Hassan II, Casablanca, Morocco; ^4^ Mohammed VI Center for Research and Innovation, Rabat, Morocco and Mohammed VI University of Sciences and Health, Casablanca, Morocco

**Keywords:** TMIGD2, glioma, costimulatory, immunotherapy, prognosis

## Abstract

**Introduction:**

Among all types of central nervous system cancers, glioma remains the most frequent primary brain tumor in adults. Despite significant advances in immunomodulatory therapies, notably immune checkpoint inhibitors, their effectiveness remains constrained due to glioma resistance. The discovery of TMIGD2 (Transmembrane and Immunoglobulin Domain Containing 2) as an immuno-stimulatory receptor, constitutively expressed on naive T cells and most natural killer (NK) cells, has emerged as an attractive immunotherapy target in a variety of cancers. The expression profile of TMIGD2 and its significance in the overall survival of glioma patients remains unknown.

**Methods:**

In the present study, we first assessed TMIGD2 mRNA expression using the Cancer Genome Atlas (TCGA) glioma transcriptome dataset (667 patients), followed by validation with the Chinese Glioma Genome Atlas (CGGA) cohort (693 patients). Secondly, we examined TMIGD2 protein staining in a series of 25 paraffin-embedded blocks from Moroccan glioma patients. The statistical analysis was performed using GraphPad Prism 8 software.

**Results:**

TMIGD2 expression was found to be significantly higher in astrocytoma, IDH-1 mutations, low-grade, and young glioma patients. TMIGD2 was expressed on immune cells and, surprisingly, on tumor cells of glioma patients. Interestingly, our study demonstrated that TMIGD2 expression was negatively correlated with angiogenesis, hypoxia, G2/M checkpoint, and epithelial to mesenchymal transition signaling pathways. We also demonstrated that dendritic cells, monocytes, NK cells, gd T cells, and naive CD8 T cell infiltration correlates positively with TMIGD2 expression. On the other hand, Mantel-Cox analysis demonstrated that increased expression of TMIGD2 in human gliomas is associated with good overall survival. Cox multivariable analysis revealed that TMIGD2 is an independent predictor of a good prognosis in glioma patients.

**Discussion:**

Taken together, our results highlight the tight implication of TMIGD2 in glioma progression and show its promising therapeutic potential as a stimulatory target for immunotherapy.

## Introduction

Since 2015, new insights into the immunology of the central nervous system (CNS) have significantly redefined the concept of its immunological privilege (1). It is now clear that the CNS has a direct lymphatic drainage pathway with the presence of various antigen-presenting cells (APCs), and is therefore accessible to different arms of the immune system (2, 3). Immune checkpoints represent a delicate network of molecules involved in co-inhibitory and co-stimulatory pathways. They regulate the immune response, enabling both effective and protective immunity (4). The clinical use of immune checkpoint inhibitors to restore a potent anti-tumor immune response has demonstrated impressive therapeutic effects, and has thus revolutionized the paradigms of cancer immunotherapy (5). In the field of CNS, specifically brain cancer, the most widespread and malignant primary tumors are gliomas. Regardless of the rigorous mainstay of therapy, the prognosis of these tumors is still largely dismal with a median survival of 14.6 months and a 5-year survival rate of only 6.8%, especially for the deadliest and most resistant form, Glioblastoma (6–8). Moreover, current immunotherapy approaches have not improved outcomes in patients with glioma. The expected increase in median overall survival (OS) was not observed in the Phase II and Phase III clinical trials of the PD-1/PD-L1 inhibitors, nivolumab or durvalumab (9, 10). On the basis of these clinical observations, there is an urgent need to develop more effective immunostimulatory strategies and to search for new biomarkers to improve response rates in gliomas (11). Along with blocking inhibitory checkpoints, fostering co-stimulatory pathways strengthens the immune system and appears to have encouraging results in cancer (12). These co-stimulatory molecules can be expressed on activated T cells, where molecules like ICOS, OX40, CD137, and GITR are present, or on naïve T cells, where molecules like CD27 and CD28 are expressed (13–16).The third class of B7-CD28 immune checkpoint family is composed of B7-H3, B7x, HHLA2, and the recently identified receptor transmembrane and immunoglobulin domain containing 2 (TMIGD2) (17, 18). Initially known as immunoglobulin-containing and proline-rich receptor-1 (IGPR-1) and CD28 homolog (CD28H),TMIGD2 is a membranous glycoprotein belonging to the immunoglobulin superfamily (IgSF) (19). Furthermore, TMIGD2 was characterized as an HHLA2 receptor that induces significant co-stimulation in human T cell responses *via* increasing cell growth and cytokine production through an AKT-dependent signaling cascade, both *in vitro* and *in vivo*. Regarding expression on immune cells, the work of Zhu and colleagues emphasized the presence of TMIGD2 proteins constitutively on naive T cells, and most of NK cells (20). On the other hand, endothelial and epithelial cells have been reported to express TMIGD2, which plays a role in inhibiting cell migration and enhancing capillary tube development during angiogenesis (21). A recent study using B7H7+ tumor cells showed that TMIGD2 is an activator of NK cells and demonstrated its promising potential as a target to enhance anti-tumor activity (22). In gastric cancer, elevated TMIGD2 expression predicts a bad prognosis, mainly when its ligand HHLA2 is strongly co-expressed (23). Conversely, in gliomas and pancreatic ductal adenocarcinomas, tumors with high levels of HHLA2 expression had a better prognosis, suggesting that it may act as a co-stimulator through the TMIGD2 pathway (24, 25). However, the expression profile of the TMIGD2 immune checkpoint and its prognostic value in gliomas remain unknown. In the present study, we assessed TMIGD2 expression levels in glioma patients, demonstrating its potential use as a target for immunotherapy.

## Materials and methods

### Patients and samples

All patients underwent initial surgery between June 2016 and August 2021 at the department of neurosurgery, Ibn Rochd University Hospital, Casablanca, Morocco. All patients have accepted surgical treatment in the hospital and have been diagnosed with glioma. A total of six patients were diagnosed with a grade 1 glioma, five with a grade 2 glioma, seven with a grade 3 glioma, and seven with a grade 4 glioma. No patient has received chemotherapy or radiotherapy before surgery. At recruitment, a consent form was signed by each subject. Clinical and pathological data were obtained from a review of the patient’s medical records.

### Immunohistochemistry

The evaluation of TMIGD2 expression by immunohistochemistry was carried out in accordance with the steps of the immunohistochemistry protocol described above (26).

The primary antibodies used in this study were rabbit polyclonal TMIGD2 (Rockland Immunochemicals, USA), Rabbit IgG Isotype control (Bioss Antibodies, USA). All paraffin-embedded tumor tissue specimens (n = 25) were collected from patients with glioma who underwent surgery at the Ibn Rochd University Hospital, neurosurgery department (Casablanca, Morocco). For positive control, paraffin blocks of the normal small intestine were collected from the Department of Pathological Anatomy at the CHU Ibn Rochd. First, the sections (5 µm thick) were incubated at 65°C for 1 hour and then overnight at 37°C. For the antigen retrieval step, sections were placed in PT Link (Dako, Denmark) bath in a high-pH retrieval solution (Envision Flex target retrieval solution, low PH (27) in 30 ml, Dako, Denmark) at 98°C for 20 min. Thereafter, the tissues were imbibed in 3% hydrogen peroxide (EnVision flex peroxidase-blocking reagent, Dako, Denmark) for 10 minutes and, then, incubated in a washing buffer (EnVision flex wash buffer, Dako, Denmark) to inhibit endogenous peroxidase activity. Next, the slides were incubated for 1 hour with the primary antibody rabbit anti human TMIGD2 antibody at a concentration of 2.5 μg/ml. For all cases, a rabbit IgG isotype control was used as a negative control at a 1:200 dilution. Subsequently, sections were incubated for 20 min at room temperature with secondary horseradish peroxidase-conjugated goat anti-rabbit anti-mouse IgG (EnVision Flex/HRP, Dako, USA). The slides were then stained with chromagen 3, 3’-diaminobenzidine (DAB) (EnVision DAB + CHROMOGEN, Dako, USA). Finally, all sections were soaked in hematoxylin for 3 minutes and dehydrated in alcohol solutions (75%, 95%, 100%), and mounted.

### Evaluation of immunostaining

Cells stained positive for TMIGD2 in glioma specimens were counted on the 40x (objective) images of immune cells and glioma cells. The staining intensity (SI) was determined as follows: 0 (negative), 1 (weak), 2 (intermediate) or 3 (strong). The percentage of positive cells (PP) was scored as follows: 0%–100%. The value of SI×PP in each section was regarded as the IHC score of the TMIGD2 expression on immune cells and glioma cells ^1^.

Pathologists were blinded to sample identity. As a positive control tissue, a small intestine was used.

### TCGA and CGGA datasets analysis

Clinical data and mRNA-seq for glioma patients were downloaded from public databases. The Cancer Genome Atlas (TCGA) dataset from https://www.cbioportal.org/contains 513 LGG samples and 154 GBM samples, ranging from WHO grades 2 to 4. As a validation cohort, we used data from the Chinese Glioma Genome Atlas (CGGA) http://www.cgga.org.cn/download.jsp) database, which includes 188 grade 2, 255 grade 3, and 249 grade 4. The clinical information included diagnosis, age, sex, cancer type, grade, IDH1 mutation, radiation therapy, vital status, and OS rates.

### The immune cell abundance identifier

The ImmuCellAI (http://bioinfo.life.hust.edu.cn/web/ImmuCellAI/) was used to assess the relative abundance of 24 immune cells subpopulations in glioma patient’s microenvironment expressing a low or high level of TMIGD2.

### Gene set enrichment analysis

A Gene Set Enrichment Analysis (GSEA) (https://software.broadinstitute.org/gsea/index.jsp) was performed using the R package “clusterProfiler” based on TCGA and CGGA datasets to determine the enriched biological processes in TMIGD2 groups. Enriched terms with a false discovery rate (FDR) < 0.05 were significant.

### Statistical analysis

All data were analyzed using GraphPad Prism 8.0 software (GraphPad Software, Inc., La Jolla, CA, USA). The overall survival (OS) according to TMIGD2 expression profile was estimated by Log-rank (Mantel-Cox) test, and t-test was used to compare means between groups. The association between TMIGD2 expression and genes of interest was assessed using Spearman’s correlation analysis. A multivariable Cox analysis was performed to estimate the association between TMIGD2 expression and patient’s prognosis. The median expression level of TMIGD2 was used to categorized all patients. For all statistical methods, p<0.05 was considered as significant difference.

### Ethics approval

A study of this project was approved by the ethical committee at the Ibn Rochd University Hospital of Casablanca. Regulations and guidlines were followed to implement the methods.

## Results

### TMIGD2 gene expression is up-regulated in low grade glioma tissues

**Figure 1:**
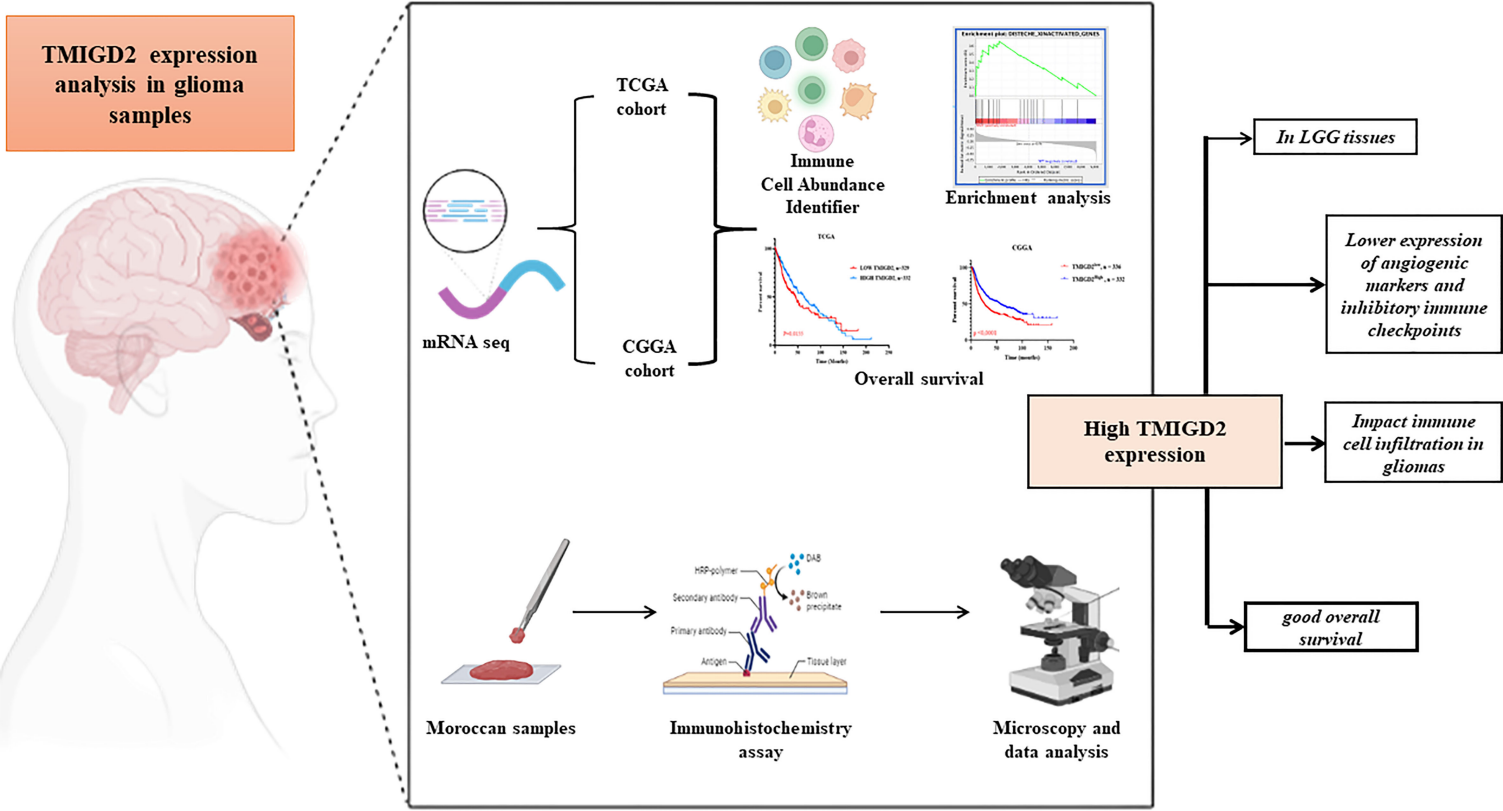
Graphical Abstract.

To assess the association between TMIGD2 gene expression and clinical features of glioma patients, we analyzed transcriptomic data from the TCGA (248 grade 2, 264 grade 3, and 153 grade 4 patients) and the CGGA cohorts (188 grade 2, 255 grade 3, and 249 grade 4 patients) (**Figure 1**). The features of all patients in both datasets were described in (**Table 1**).

**Table 1:** The clinicopathological features of glioma patients.

Caracteristic	Training cohort TCGA Cases (%) (n = 667)	Validation cohort CGGA Cases (%) (n = 693)
Age (years)		
> 45	**314 (47.08)**	382 (55.13)
45 ≤	**305 (45.72)**	310 (44.73)
Missing data	**48 (7.2)**	1 (0.14)
Sex		
Male	**348 (52.17)**	398 (57.43)
Female	**271 (40.63)**	295 (42.57)
Missing data	**48 (7.2)**	0
WHO Grade		
Low grade (II)	**248 (37.18)**	188 (27.13)
High grade (III-IV)	**417 (62.52)**	504 (7273)
Missing data	**2 (0.3)**	1 (0.14)
Histological Subtypes		
Astrocytomas	**194 (29.08)**	271 (39.11)
Oligoastrocytomas	**130 (19.50)**	14 (2.02)
Oligodendrogliomas	**189 (28.33)**	158 (22.80)
Glioblastomas	**153 (22.94)**	249 (35.93)
Missing data	**1 (0.15)**	1 (0.14)
IDH1 Mutation		
Mutant	**421 (63.12)**	356 (51.37)
Wild-type	**202 (30.28)**	286 (41.27)
Missing data	**44 (6.6)**	51 (7.36)

% Percentage of case.

The values in bold are the number of cases, and the values in brackets are their percentage.

**Figure 2:**
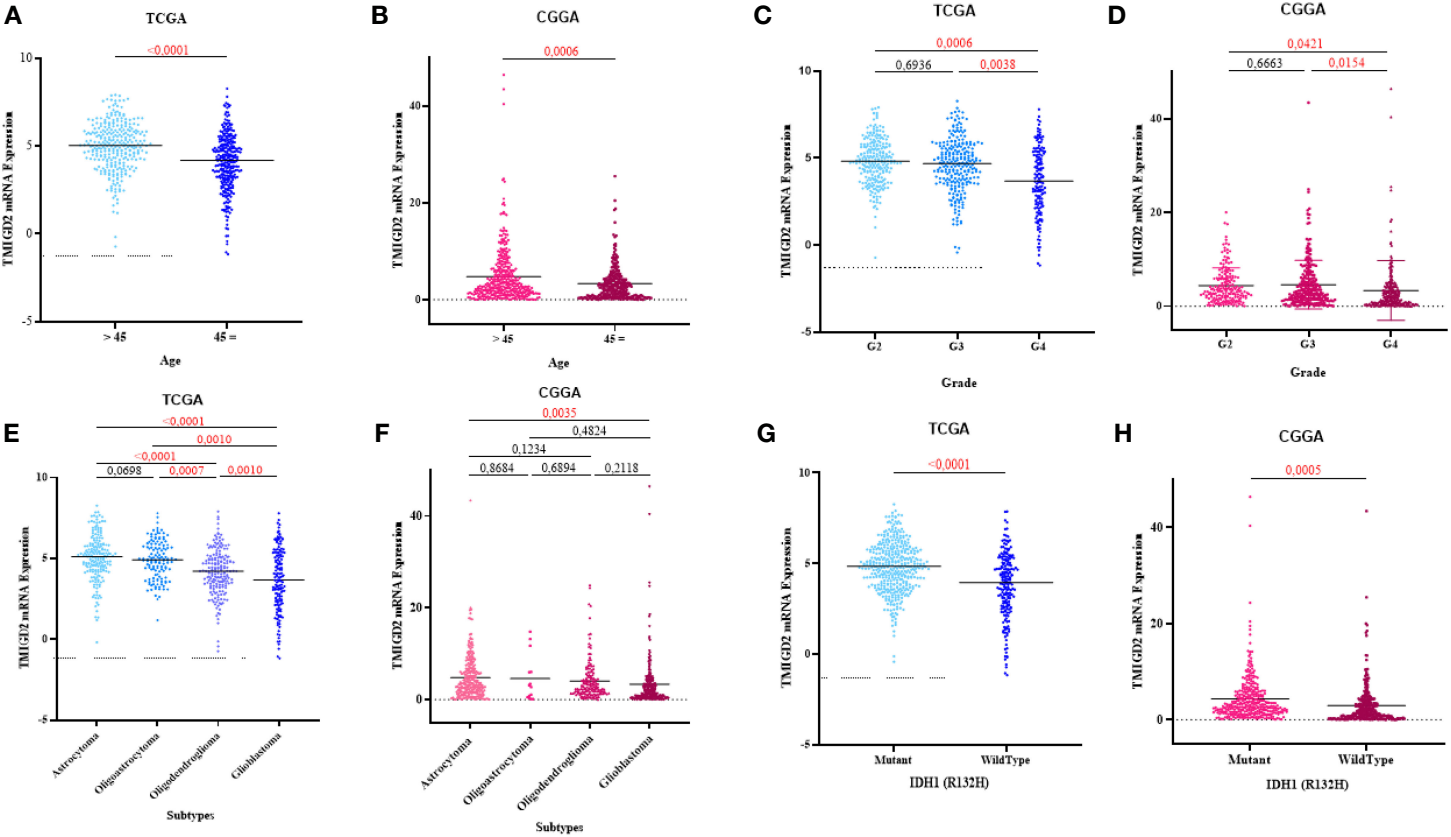
Association between TMIGD2 expression and clinical parameters of glioma in the TCGA and CGGA cohorts. TMIGD2 expression was significantly increased in young glioma patients **(A, B)**. TMIGD2 was highly expressed in grade 2 gliomas at the transcription level compared with grade 3 gliomas and glioblastoma (GBM, WHO grade 4) **(C, D)**. The astrocytoma group had a higher expression of TMIGD2 in TCGA and CGGA datasets **(E, F)**. TMIGD2 was enriched in IDH1 mutant glioma **(G, H)**.

TMIGD2 expression was significantly higher in young patients than older (p< 0.0001) (**Figures 2A, B**). As compared to grade 2 and grade 3 gliomas, glioblastomas expressed a lower level of TMIGD2 transcript (p= 0.0006; p= 0.0038) (**Figures 2C, D**). Besides, TMIGD2 expression was upregulated in the astrocytoma subtype compared to glioblastoma (p< 0.0001) (**Figures 2E, F**). In the TCGA group, oligoastrocytomas and oligodendrogliomas displayed a high level of TMIGD2 than the glioblastomas subtype (p= 0.0010; p= 0.0010). Further, mutant IDH-1 patients exhibit significantly higher levels of TMIGD2 than IDH-1 wild-type patients (p< 0.0001) (**Figures 2G, H**). These results were well validated in the CGGA cohort.

To corroborate the results of TMIGD2 expression obtained in the transcript level (**Figure 1**), TMIGD2 protein analysis was performed on 25 glioma samples (6 Grade I, 5 Grade II, 7 Grade III, and 7 Grade 4) by immunohistochemistry (**Table 2**).

**Table 2:** Glioma patients’ characteristics.

Moroccan Cohort Variable	Cases (%) (n = 25)
Age (years)	
<48	17 (68)
≥47	8 (32)
Missing data	0 (0)
Sex	
Male	15 (60)
Female	10 (40)
Missing data	0 (0)
WHO Grade	
Low grade (I-II)	11 (44)
High grade (III-IV)	14 (56)
Missing data	0 (0)
Histological Subtypes	
Astrocytomas	7 (28)
Astroblastomas	2 (8)
Oligoastrocytomas	2 (8)
Oligodendrogliomas	4 (16)
Ependymomas	3 (12)
Glioblastomas	7 (28)
Missing data	0 (0)
IDH1 Mutation	
Mutant	9 (36)
Wild-type	13 (52)
Missing data	3 (12)

%Percentage of case.

**Figure 3:**
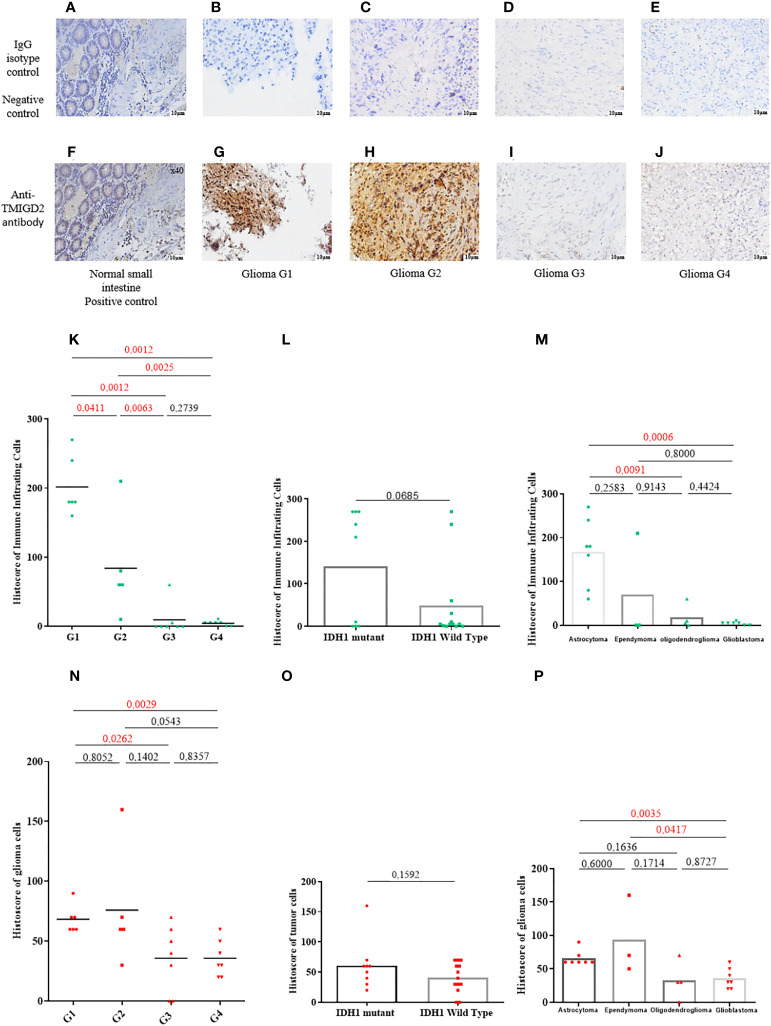
TMIGD2 expression in primary human glioma samples (magnification 40×). Negative control staining of normal small intestine tissue using a rabbit IgG isotype control (magnification 40×) **(A)**. Negative control staining of glioma patients (WHO 1 grade glioma to WHO 4 grade glioma) with a rabbit IgG isotype control **(B–E)** (magnification 40×). Positive TMIGD2 staining in the normal small intestine (magnification 40×) **(F)**. Glioma tissues of different grades (WHO 1 grade glioma to WHO 4 grade glioma) were immunostained for TMIGD2 using anti-TMIGD2 antibody and 3,30-diaminobenzidine (DAB; brown) **(G–J)** (magnification40×). Histoscores of immune infiltrating cells for TMIGD2 staining in different grades of glioma**(K)**, IDH status **(L)**, and histological subtypes **(M)**. Histoscores of TMIGD2 staining on glioma cells according to WHO grades of glioma **(N)**, IDH status **(O)**, and histological subtypes **(P)**.

The normal small intestine has been reported to constitutively express the TMIGD2 protein (21). When the IgG was used as a negative control, neither the small intestine (**Figure 3A**) nor the glioma samples stained (**Figures 3B–E**). TMIGD2 staining was observed in the normal small intestine tissue once an anti-TMIGD2 mAb was applied under identical circumstances (positive control) (**Figure 3F**). TMIGD2 protein staining was higher in glioma grades 1 and 2 (**Figures 3G, H**) as opposed to grades 3 and 4 (**Figures 3I, J**). Interestingly, TMIGD2 protein expression was upregulated on infiltrating immune cells and tumor cells in LGG patients (p= 0.0012; p= 0.0029) (**Figures 3K, N**). Additionally, neither immune nor tumor cells with wild-type or muted IDH-1 displayed statistical differences in TMIGD2 expression (p= 0.0685; 0.1592) (**Figures 3L, O**). Moreover, our results showed that astrocytomas are enriched with immune cells expressing high levels of TMIGD2, unlike oligodendrogliomas and glioblastomas subtypes (p= 0.0091; p= 0.0006) (**Figure 3M**). On tumor cells, however, TMIGD2 is more highly expressed on ependymoma and astrocytoma subtypes than on glioblastoma (p= 0.0417; p= 0.0035) (**Figure 3P**). Collectively, these findings indicate that the TMIGD2 molecule is associated with less aggressive clinicopathological characteristics in glioma patients.

### TMIGD2 is negatively correlated with angiogenic markers

**Figure 4:**
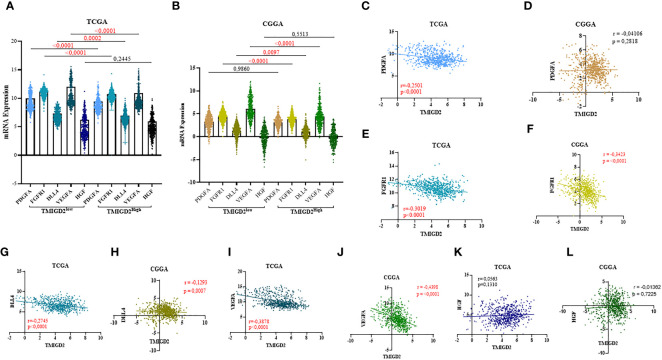
Correlation between TMIGD2 and angiogenic markers in TCGA and CGGA datasets. PDGFA, FGFR1, DLL4 and VEGFA, representative markers of angiogenesis, were significantly higher in the low-TMIGD2 expression group **(A, B)**. TMIGD2 was negatively correlated with angiogenesis molecules, including PDGF **(C, D)**, FGFR1 **(E, F)**, DLL4 **(G, H)** and VEGFA **(I, J)**, and no significant association was found between the expression of TMIGD2 and HGF in glioma TME **(K, L)**.

**Figure 5:**
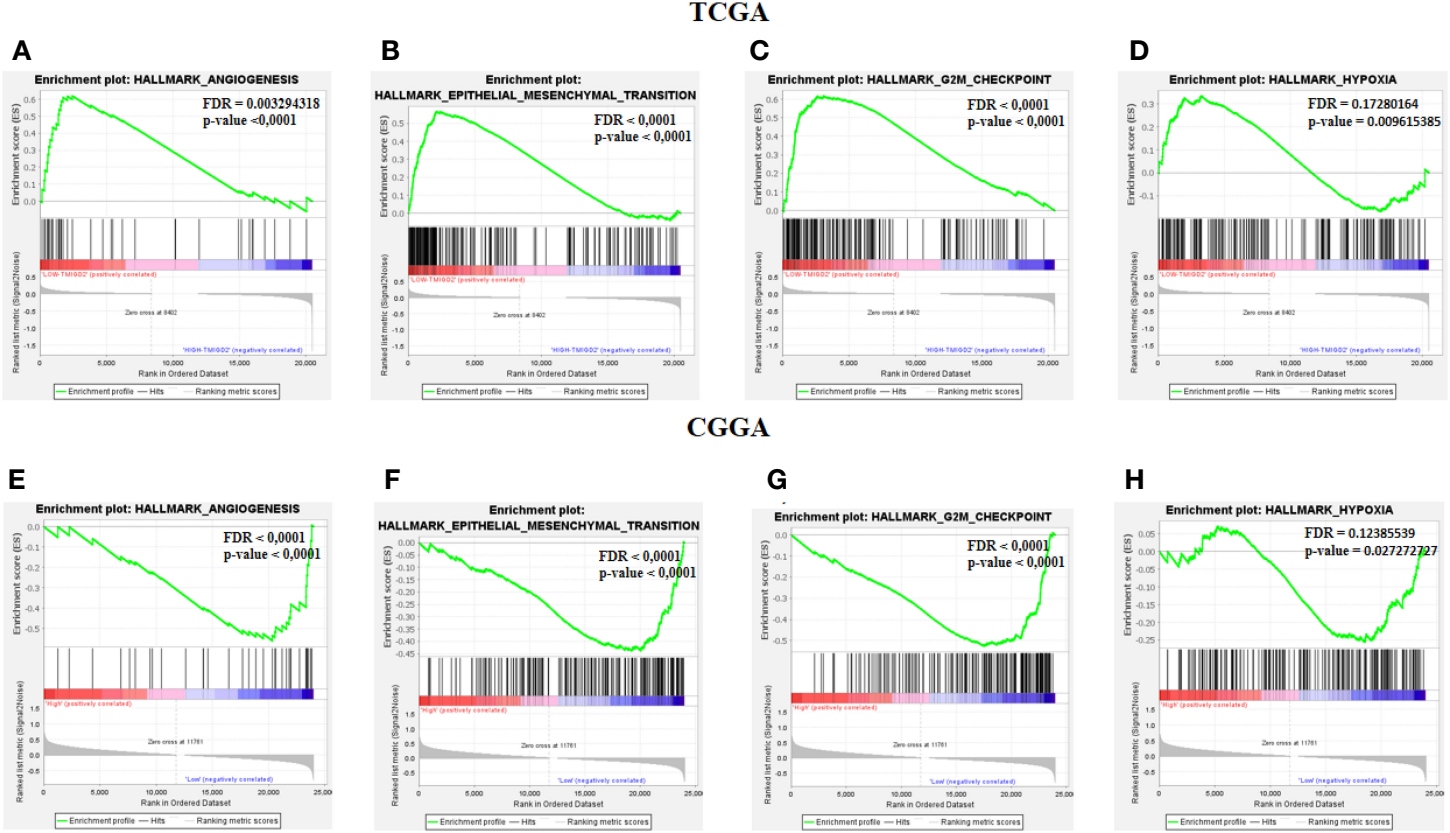
Gene Set Enrichement analysis. Enrichment analysis between low and high TMIGD2 groups shows the angiogenesis **(A, F)**, epithelial mesenchymal transition **(B, G)**, G2M checkpoint **(C, H)**, and hypoxia **(D, I)**, genes signaling pathway.

After characterizing the expression pattern of TMIGD2 according to clinical parameters in gliomas, we next investigated the association between TMIGD2 and angiogenesis markers. As illustrated in (**Figures 4A, B**) Fibroblast Growth Factor Receptor (FGFR), Delta Like Ligand 4 (DLL4), and Vascular Endothelial Growth Factor (VEGF) were significantly higher in a group of patients with low TMIGD2 (p<0.0001; p= 0.0002; p< 0.0001). According to the TCGA cohort, we found a weak negative correlation between TMIGD2 expression and the pro-angiogenic factors including platelet-derived growth factor (PDGFA) (r= -0.2501; p< 0.0001), hepatocyte growth factor (HGF) (r= -0.4398; p< 0.0001), and DLL4 (r= -0.2745; p< 0.0001), (**Figures 4C, D, G, H, K, L**). However, TMIGD2 performed a negative correlation with vascular endothelial growth factor (VEGF) (r= -0.3878; p< 0.0001), and fibroblast growth factor receptor 1 (FGFR1) (r= -0.3019; p< 0.0001) (**Figures 4E, F, I, J**). On the other hand, different studies have reported that epithelial to mesenchymal transition, hypoxia, and G2/M checkpoint signaling pathways promote angiogenesis (28–30). Therefore, gene set enrichment analysis (GSEA) was performed to explore the impact of TMIGD2 in these pathways. We have found that genes associated with angiogenesis (FDR= 0.00329; p<0.0001), epithelial to mesenchymal transition (FDR<0.0001; p<0.0001), hypoxia (FDR= 0.1728; p= 0.009), and G2/M checkpoint (FDR<0.0001; p<0.0001) signaling pathways were significantly enriched in patients with low TMIGD2 expression (**Figures 5A–D**). Similar results were reported in the CGGA cohort (**Figures 5F–I**). According to the above data, TMIGD2 expression decreases with glioma progression pathways, suggesting that TMIGD2 may prevent glioma development.

### TMIGD2 transcripts negatively correlated with inhibitory immune checkpoints

**Figure 6:**
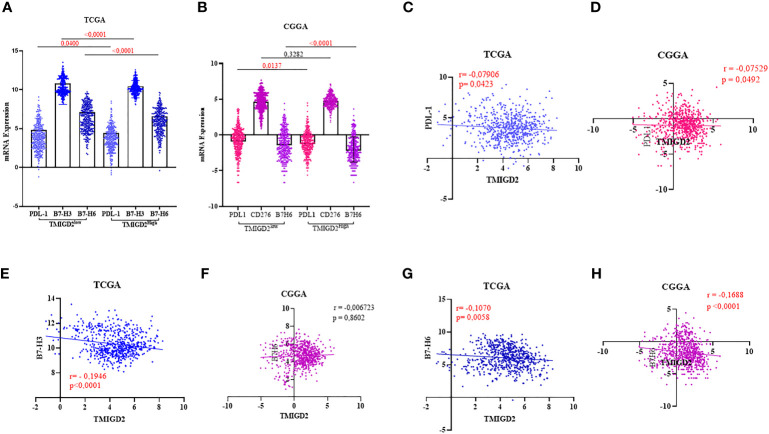
Association between TMIGD2 and inhibitory immune checkpoints in human gliomas from the TCGA and CGGA cohorts. PDL1, B7H3 and B7H6 were significantly higher in the low-TMIGD2 expression groups **(A, B)**. TMIGD2 expression negativelycorrelated with PDL1 **(C, D)**, and B7H6 **(G, H)**. Weak negative correlation between TMIGD2 and B7H3 in TCGA cohort **(E)**. There is no significant correlation between TMIGD2 and B7H3 in patients in the CGGA database **(F)**.

Using the same cohorts of patients (TCGA and CGGA), we assessed the association of TMIGD2 expression with inhibitory immune checkpoints that belong to the b7-family members. These immune check points Programmed death-ligand 1 (PDL-1), B7-H3 (CD276), and B7-H6 (NCR3LG1) have been associated with aggressiveness and poor prognosis in glioma patients (31–33). Interestingly, the expression of inhibitory immune checkpoints was significantly upregulated in patients with a low TMIGD2 expression profile (**Figures 6A, B**). Nevertheless, the Pearson correlation showed a weak negative correlation of TMIGD2 with PDL-1(r=-0.07906; p= 0.0423), (r=-0.07529; p= 0.0492) (**Figures 6C, D**) and B7-H6 (r= -0.1070; p= 0.0058), (r= -0.1688; p < 0.0001) (**Figures 6G, H**). In addition, a weak negative correlation between TMIGD2 and B7H3 was found in the TCGA cohort (**Figure 6E**), while no significant correlation between TMIGD2 and B7H3 was observed in patients in the CGGA database (**Figure 6F**). Our results showed that TMIGD2 was not correlated with the expression of B-7 family immune checkpoints.

### TMIGD2 expression levels affect immune cell infiltration in gliomas

**Figure 7:**
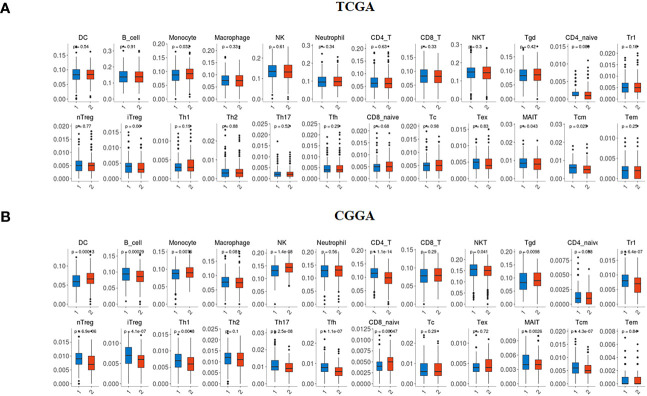
Immune infiltration profile according to TMIGD2 expression in glioma patients. In the TCGA **(A)** and CGGA **(B)** cohorts, blue boxes represent the abundance of immune cell subsets in low TMIGD2 group, and red boxes correspond to the infiltration rate in high TMIGD2 group.

We collected data from two databases (TCGA and CGGA) to examine the abundance of 24 immune cell subsets based on the TMIGD2 profiles. In the TCGA dataset, Monocytes appeared more abundant in patients with high TMIGD2 (p=0.032), whereas, the infiltration level of Mucosal-Associated Invariant T cells (MAIT) (p= 0.043) and central memory T cells (Tcm) (p= 0.029) were less abundant (**Figure 7A**). In the CGGA cohort, patients with high TMIGD2 profile showed high infiltration levels of dendritic cells (DC) (p= 0.00013), monocytes (p= 0.0076), NK (p<0.0001), γδ T cells (Tgd) (p= 0.0098), MAIT (p= 0.0026), and naive CD8 T cells (p= 0.00047). In contrast, B cells (p= 0.00029), CD4 T (p<0.0001), natural killer T (NKT) cells (p= 0.041), type 1 regulatory T cell (Tr1) (p<0.0001), Natural regulatory T cells (nTregs) (p<0.0001), induced T regulatory cells (iTreg) (p<0.0001), T helper type 1 (Th1) (p= 0.0048), T helper type 17 (Th17) (p<0.0001), T follicular helper cells (Tfh) (p<0.0001), and Tcm (p<0.0001) were less infiltrated (**Figure 7B**). Overall, our study suggests that TMIGD2 expression was positively associated with anti-tumor immune cell infiltration, while negatively associated with pro-tumor immune cell subsets.

### Elevated expression of TMIGD2 in the microenvironment of glioma patients is associated with good OS

**Figure 8:**
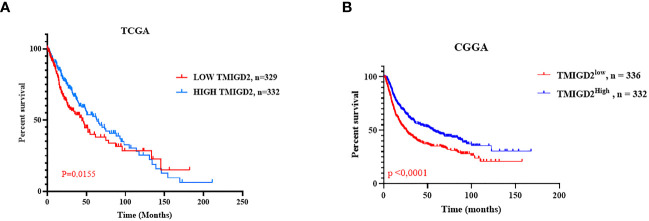
Overall survival in the TCGA and CGGA cohorts according to TMIGD2 expression in the glioma tumor microenvironment. Blue curves represent the OS of low TMIGD2 patients. Red curves reflect the OS of high TMIGD2 patients **(A, B)**.

**Figure 9:**
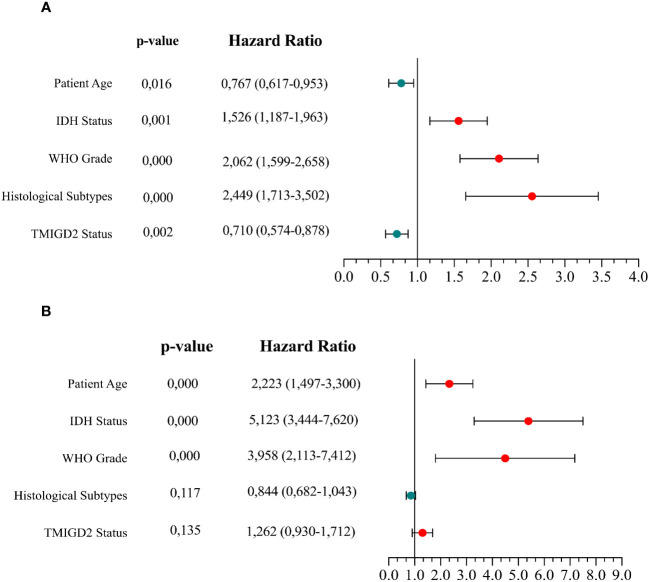
Cox multivariable analysis of clinical prognostic characteristics in the TCGA and CGGA datasets. Cox analyses investigating the variables for survival of glioma patients in the TCGA cohort **(A)** and CGGA cohort **(B)**.

To establish whether the difference in TMIGD2 expression would reliably affect the outcome of patients, we performed Mantel-Cox analysis between low and high TMIGD2 groups. As a matter of fact, patients who presented high expression levels of TMIGD2 were strikingly associated with a good outcome (at 50% of OS, the survival median was at roughly 70 months) (p=0.0155) compared to those with low expression levels, which fits our results in (**Figures 8A, B**). In addition, multivariable analysis indicated that TMIGD2 and age were independent good prognostic biomarkers in the TCGA dataset (HR= 0.710; p=0.002; HR= 0.767; p=0.016) respectively (**Figure 9A**). However, WHO grade (HR= 2.062; p<0.0001), histological subtype (HR= 2.449; p<0.0001), and IDH Status (HR= 1.526; p= 0.001) were independent of worse prognosis biomarkers in both glioma cohorts (**Figures 9A, B**). These results indicate that TMIGD2 is associated with a better prognosis in human gliomas.

## Discussion

Glioblastoma is the most aggressive type of glioma, and its proximity to sensitive areas of the brain makes surgical resection clinically arduous (34). Furthermore, conventional approaches to dealing with recidivism are inefficient (35), hence the need to explore new treatment strategies. Immunotherapy is a well-known field of cancer treatment method that involves using the immune system to combat cancer cells (36). Numerous studies have evaluatZSed the expression of inhibitory receptors on exhausted CD8^+^T cells, yet costimulatory molecules are still poorly explored (37). To the best of our knowledge, the present study is the first work to evaluate the expression of TMIGD2, and its association with clinical characteristics, angiogenesis markers and OS in glioma patients. Using the TCGA, the CGGA, and the Moroccan cohorts, we characterized the expression profile of TMIGD2 at the transcriptional and proteomic levels in diffuse gliomas. TMIGD2 protein staining results showed an upregulation on both tumor and immune cells in LGG versus HGG patients. This study is the first to characterize TMIGD2 expression on human glioma cells. Yet, its role on these cells is still unknown. In gastric cancer, immunohistochemistry analysis reported that TMIGD2 protein was expressed in GC and noncancerous samples, and on immune cells in both tissues. Furthermore, TMIGD2 expression on immune cells was not significantly associated with pathological grade in GC. In high grade glioma, a previous study showed a lack of naive T cells in the tumor microenvironment (TME) (38). Besides, some studies targeting NK cells have shown that they are more abundant in low-grade gliomas than in high-grade gliomas (39).Given that TMIGD2 is constitutively expressed on naive T cells and most NK cells (20), this may explain the low level of TMIGD2 in HGG tissue. In terms of patient age, our analysis showed that TMIGD2 expression is highly abundant in TME of young patients (≤ 45). In fact, the number of NK cells and their proliferative capacity decline with aging (40, 41). Furthermore, the production of naïve T cells, the repertoire of T cell receptors, and T cell activation are all negatively impacted by a patient’s age, which provides an explanation for this observation (42, 43). TCGA cohort analysis revealed a significant expression of TMIGD2 in the IDH1 mutation group. Prior findings have indicated that patients with IDH mutant status have prolonged OS compared to those with wild-type status (44–47). In addition, high TMIGD2 protein staining on immune cells was significantly associated with the astrocytoma subtype. In contrast to tumor cells, TMIGD2 protein staining was strongly associated with ependymoma subtype. It has been reported that glioblastoma multiform (GBM) was associated with a worse prognosis than astrocytoma and ependymoma subtypes (48, 49). These results demonstrate that overexpression of TMIGD2 was associated with the lower-grade IDH1-mutant tumors and may be correlated with a better outcome in patients with gliomas. This disease represents the most highly vascularized malignancy, and severe angiogenesis is one of its distinctive pathological features (27). Nader and colleagues’ study shed light on the implication of TMIGD2 in cell-cell interaction, cell migration, and angiogenesis. The angiogenic phenotype of endothelial cells in culture was considerably influenced by the ectopic expression or silencing of TMIGD2 expression, and the introduction of TMIGD2 to tumor cells promoted tumor angiogenesis *via* the SPIN90/WISH signaling axis (21). Moreover, several studies have shown the strong involvement of hypoxia, the epithelial to mesenchymal transition, and G2/M checkpoint signaling pathways in mediating angiogenesis (28–30). As part of our study, we assessed the association between TMIGD2 expression and various genes associated with angiogenesis and tumor progression. Interestingly, we observed a significantly lower expression of genes such as PDGFA, FGFR1, DLL4, and VEGFA in glioma patients with a higher TMIGD2 profile. Furthermore, Gene Set Enrichment Analysis revealed that angiogenesis-associated genes were mainly enriched in patients with low TMIGD2 expression. Qi Y and colleagues’ study reported that overexpression of TMIGD2 ligand may inhibit angiogenesis in gliomas through anti-VEGF and anti-PDGF processes (25). Accordingly, our results suggest that high TMIGD2 expression may be associated with low angiogenesis potential and thus with a lower risk of tumor development and metastasis. Otherwise, therapies targeting TMIGD2 may not only improve antitumor immune responses, but also affect tumor angiogenesis.In recent years, exciting progress has been made in the use of ICI in several solid tumors (50, 51). Over the past few years, there have been a growing number of studies on molecules in the B7 family, specially B7-H3, B7-H4, B7-H5, B7-H6, and B7-H7 (31). In glioma tissues, it has been reported that high expression of inhibitory molecules, namely PDL1, B7H3 and B7H6 promotes tumor progression (31–33). Previous studies have demonstrated that PDL1, B7H3, B7H6 blockade enhance naïve T cell priming in draining lymph nodes, reactivating dysfunctional T cells in tumor tissues, or by increasing antitumor response (52–55). Further, PDL1 inhibition induces the expansion of CD4/CD8 T cells expressing ICOS molecules in the tumor microenvironment of colorectal cancer patients (56). Considering that TMIGD2 belongs to the B7 family, our study is the first to examine the association of TMIGD2 expression with PD-L1, B7H3, and B7H6 in gliomas. We report here that high TMIGD2 expression was negatively associated with PDL1, B7H3 and B7H6 expression compared to low TMIGD2 profile. Our results suggest that high TMIGD2 expression may be involved in the anti-tumor immune response and in the inhibition of glioma development. On the other hand, tumor infiltrating immune cells have been reported to predict the treatment response and prognosis of glioma patients (39). Therefore, the association between TMIGD2 and immune cell infiltration was investigated. Our results showed that dendritic cells, monocytes, NK cells, γδ T cells, and naive CD8 cells were abundant in the TME of patients with high TMIGD2 expression. In addition, infiltration of B cells, CD4 T cells, NKTs, Tr1s, nTregs, iTregs, Th1s, Th17s, Tfhs, and Tcms was reduced in gliomas with high TMIGD2 levels. Indeed, many studies have found an association between long-term survival in glioma patients and dendriticcells, NK cells, γδ T cells, and CD8 cells infiltration (57–59). In contrast, CD4 T cells, Tr1s, nTregs, iTregs, Th1s, and Th17s infiltrating the glioma tumor microenvironment were correlated with immunosuppression and a poor prognosis (6, 60–62). Our findings suggest that overexpression of TMIGD2 may promote infiltration of antitumor immune cell subsets in the glioma TME. Regarding the OS analysis, our findings indicated that glioma patients with high TMIGD2 expression presented an improved outcome. Additionally, TMIGD2 represents an independent predictor of a good prognosis in glioma patients. This observation was also reported in pancreatic ductal adenocarcinoma, where the HHLA2/TMIGD2 pathway is associated with a better prognosis (24). Contrary to these results, patients with negative HHLA2^/^TMIGD2 expression presented a significantly higher 5-year OS compared to the positive groups of gastric cancer (23). In dysplasia and oral squamous cell carcinoma, elevated levels of both HHLA and TMIGD2 also demonstrated a poor prognosis (63). Our current data shows for the first time that a high TMIGD2 expression profile displays a favorable outcome in patients with gliomas. Overall, TMIGD2 could be a potential therapeutic target associated with improved OS in glioma patients. Low TMIGD2 expression could predict worse clinical characteristics, angiogenesis, and protumoral immune cell infiltration. More importantly, TMIGD2 may be a relevant biomarker for patient selection and for response to immunotherapy evaluation in gliomas. Considering that naive T-cell deficiencies at diagnosis and after chemotherapy impair cell therapy potential (64). Further, bevacizumab (anti-VEGF) did not show an overall survival advantage in glioma patients (65). We, hence propose combining anti-VEGF treatment and selective activation on naive T cells in patients with high TMIGD2 to enhance anti-tumor response and prognosis in glioma patients.

## Data availability statement

The datasets presented in this study can be found in online repositories. The names of the repository/repositories and accession number(s) can be found in the article/supplementary material.

## Ethics statement

The studies involving human participants were reviewed and approved by the ethical committee at the Ibn Rochd University Hospital of Casablanca. The patients/participants provided their written informed consent to participate in this study.

## Author contributions

CB collected, analyzed, and interpreted data, and wrote the manuscript. SA and HB collected and analyzed data. IR, MK and AL analyzed and reviewed the manuscript. AB analyzed the data, revised the manuscript and supervised the study. All authors contributed to the article and approved the submitted version.
